# Neuropathological Aspects of SARS-CoV-2 Infection: Significance for Both Alzheimer’s and Parkinson’s Disease

**DOI:** 10.3389/fnins.2022.867825

**Published:** 2022-05-03

**Authors:** Jaime Silva, Felipe Patricio, Aleidy Patricio-Martínez, Gerardo Santos-López, Lilia Cedillo, Yousef Tizabi, Ilhuicamina Daniel Limón

**Affiliations:** ^1^Laboratorio de Neurofarmacología, Facultad de Ciencias Químicas, Benemérita Universidad Autónoma de Puebla, Puebla, Mexico; ^2^Facultad de Ciencias Biológicas, Benemérita Universidad Autónoma de Puebla, Puebla, Mexico; ^3^Laboratorio de Biología Molecular y Virología, Centro de Investigación Biomédica de Oriente, Instituto Mexicano del Seguro Social, Atlixco, Mexico; ^4^Centro de Detección Biomolecular, Benemérita Universidad Autónoma de Puebla, Puebla, Mexico; ^5^Department of Pharmacology, Howard University College of Medicine, Washington, DC, United States

**Keywords:** SARS-CoV-2, Alzheimer’s disease, Parkinson’s disease, neuroinvasive pathways, blood-brain barrier

## Abstract

Evidence suggests that SARS-CoV-2 entry into the central nervous system can result in neurological and/or neurodegenerative diseases. In this review, routes of SARS-Cov-2 entry into the brain *via* neuroinvasive pathways such as transcribrial, ocular surface or hematogenous system are discussed. It is argued that SARS-Cov-2-induced cytokine storm, neuroinflammation and oxidative stress increase the risk of developing neurodegenerative diseases such as Alzheimer’s disease and Parkinson’s disease. Further studies on the effects of SARS-CoV-2 and its variants on protein aggregation, glia or microglia activation, and blood-brain barrier are warranted.

## Introduction

On December 12, 2019, in Wuhan City, Hubei Province, China, the first cases of an unexplained pneumonia failing to respond to the standard treatment regimen led to an exhaustive search for a new virus. The clinical symptoms of the condition were fever, dry cough, sore throat, pneumonia, severe dyspnea, and myalgia (Huang C. et al., 2020; Zhu N. et al., 2020). Subsequently, on February 11, 2020, the new coronavirus was identified and was termed severe acute respiratory syndrome coronavirus 2 (SARS-CoV-2). Coincidentally, a day earlier, the first draft genome of the virus was made publicly available. This enabled research groups to develop different molecular diagnostics such as RT-PCR and immunological assays. Later, CRISPR-based assays, nucleic acid microarray assays, and next generation sequencing were added (Habibzadeh et al., 2021).

The World Health Organization (WHO) is responsible for declaring a pandemic. WHO monitors disease activity on a global scale through a network of centers located in countries worldwide and has a pandemic preparedness plan that consists of six phases of pandemic alert. Phase 1 represents the lowest level of alert and usually indicates that a newly emerged or previously existing virus is circulating among animals, with low risk of transmission to humans. Phase 6, the pandemic phase, is declared when an outbreak is characterized by globally widespread and sustained disease transmission among humans (Rogers, 2022). Since by the end of February 2020, COVID-19 had already registered 83,652 cases globally (Wassie et al., 2020; Carvalho et al., 2021). On March 11, 2020, WHO declared COVID-19 a pandemic.

Patients who have concomitant comorbidities and patients admitted to the intensive care unit are significantly more likely to develop complications from COVID-19 (Sanyaolu et al., 2020). Elderly and seriously ill patients with a clinical history of cardiovascular, liver, and/or kidney disease carry the highest risk of mortality. Obesity is also a risk factor for all ages (Huang Y. et al., 2020), and age, sex, ethnicity, socioeconomic group, and geographical location may also influence the outcome (Harwood et al., 2022).

**FIGURE 1:**
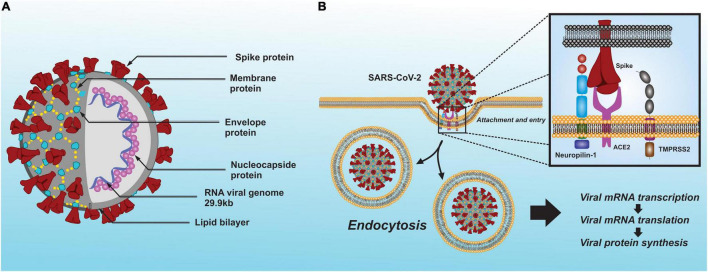
Schematic model of the SARS-CoV-2 whit its structural proteins. **(A)** The virus envelope is associated with four structural proteins: membrane protein (M); spike protein (S); envelope protein (E); and nucleocapsid protein (N). Moreover, it has a single-stranded positive sense RNA and a genome approximately 29.9 kb in size. **(B)** To enter a host cell, the SARS-CoV-2 spike protein interacts with the ACE2 and neuropilin-1, while TMPRSS2 to activate its membrane-fusion capacity and subsequent endocytosis.

The SARS-CoV-2 belongs to the *Coronaviridae* family of the genus *betacoronavirus* (βCoV) and was identified as the etiological agent of COVID-19. SARS-CoV-2, like other known coronaviruses, is an enveloped virus with single-stranded positive sense RNA and a genome approximately 29.9 kb in size (Masters, 2006; Rastogi et al., 2020). Genetically, SARS-CoV-2 and severe acute respiratory syndrome coronavirus (SARS-CoV) both have characteristically high homologous sequence, unlike the Middle East Syndrome (MERS)-CoV virus (Yu et al., 2020). The SARS-CoV-2 envelope is associated with four structural proteins: membrane protein (M); spike protein (S); envelope protein (E); and nucleocapsid protein (N) (Lu R. et al., 2020; Figure 1). Details on the structure and molecular biology of the SARS-CoV-2 virus have been recently reviewed by several groups (Arya et al., 2021; Hu et al., 2021; Peng et al., 2021; Rahimi et al., 2021).

Analysis of mutations in the coding and non-coding regions, genetic diversity, and pathogenicity of SARS-CoV-2 has also been carried out and based on the results it was suggested that a minimal variation in the genome sequence of SARS-CoV-2 may be responsible for a drastic change in the structures of target proteins, making available drugs ineffective (Naqvi et al., 2020). Clinical data show that the main structures of the body that are affected by COVID-19 are the respiratory and cardiovascular systems (Kapoor, 2020; Lazzeri et al., 2020; Yi et al., 2021). However, SARS-CoV-2 is able to infect other systems, such as the digestive, urogenital and nervous systems (Zhang Y. et al., 2020; Spudich and Nath, 2022). In this review, we discuss the recent reports on the infectivity of SARS-CoV-2 in the central nervous system (CNS) and the probable impact of COVID-19 on neurodegenerative diseases.

### Neurological Consequences of COVID-19

There are reports of COVID-19 exerting neurological effects, the most common being hypogeusia (diminished sense of taste) and anosmia (loss of sense of smell) (Gilani et al., 2020; Spudich and Nath, 2022), and cerebrovascular damage (Beyrouti et al., 2020; Meegada et al., 2020; Gilpin et al., 2021). However, encephalopathies (Helms et al., 2020), demyelination (Domingues et al., 2020; Zanin et al., 2020), edema and symptom presentations similar to multiple sclerosis and Guillain-Barré have also been observed with COVID-19 (Toscano et al., 2020; Spudich and Nath, 2022). Other neurological/neuropsychiatric symptoms such as alterations in consciousness and hallucinations in COVID-19 have been attributed to SARS-CoV-2’s effect on the frontal lobe cortex, an area intimately involved in perception (Ellul et al., 2020; Ferrando et al., 2020; Paniz-Mondolfi et al., 2020).

Because COVID-19 is a relatively recent disease, its full pathogenic mechanisms and possible sequelae in the nervous system remain unclear. Fortunately, novel molecular biology techniques, such as RT-PCR, RT-qPCR, CRISPR-based assays, and nucleic acid microarray assays have made it possible to elucidate some general aspects of the disease (Habibzadeh et al., 2021) and relate them to other public health emergencies caused by other coronaviruses, such as SARS in 2002–2003 and MERS in 2012 (Zhu Z. et al., 2020). Researchers have been able to ascertain complications associated with pre-existing conditions and the key role played by the immune system in resolution or further complication of the disease (de Wit et al., 2016; Abdelrahman et al., 2020; Ansariniya et al., 2021). In addition, SARS-CoV-2-induced acute and long-term neurological effects are a subject of intense investigation and a main focus of this article.

## Neuroinvasive Mechanisms of Severe Acute Respiratory Syndrome Coronavirus 2

Human coronaviruses not only cause common colds but can also infect neural cells as evidenced by neurotropism and neuroinvasion (Arbour et al., 2000). Studies carried out on brain samples taken from patients with SARS disease detected the presence of the SARS-CoV virus in nervous tissue (Ding et al., 2004; Xu et al., 2005). Moreover, SARS-CoV-2 has been detected in the brain and cerebrospinal fluid of COVID-19 patients using RT-qPCR and immunohistochemistry techniques (Domingues et al., 2020; Huang Y. H. et al., 2020; Moriguchi et al., 2020; Liu J. M. et al., 2021; Serrano et al., 2021; Song et al., 2021). Although the exact mechanism of neurological complications in COVID-19 patients is unknown, it has been shown that infection with SARS-CoV-2 damages the choroid plexus epithelium, leading to leakage across the blood brain barrier (Pellegrini et al., 2020). Nonetheless, potential mechanism (s) of SARS-CoV-2 entry into the CNS are a subject of intense relevance and interest (Hu et al., 2020).

It is known that both SARS-CoV and SARS-CoV-2 occupy the primary receptor angiotensin-converting enzyme 2 (ACE2) (Li et al., 2003; Lu R. et al., 2020) and can form a complex with other cofactors such as transmembrane serine protease 2 (TMPRSS2) (Hoffmann et al., 2020) and neuropilin-1 (Cantuti-Castelvetri et al., 2020; Daly et al., 2020). This interaction between SARS-CoV-2 and ACE2 is essential for the complex to be internalized into the cells (Figure 1). TMPRSS2 is vital for SARS-CoV-2 infection, although it has a low expression in the brain. However, SARS-CoV-2 can also infect cells *via* neuropilin-1 and furin protease which have a higher and broader expression in the brain compared to TMPRSS2 or ACE2 (Davies et al., 2020). Moreover, SARS-CoV-2 is likely to infect glutamatergic neurons due to higher expression of ACE2, neuropilin-1 and furin protease than GABAergic neurons (Dobrindt et al., 2021). Thus, other proteins could be involved in SARS-CoV-2 entry into the brain. Indeed, a recent study suggests that SARS-CoV-2 may interact with metabotropic glutamate receptor 2 (mGluR2), which may play a role in internalization and perhaps in SARS-CoV-2 neurotropism (Wang J. et al., 2021).

ACE2 is highly expressed in adipose tissue and organs such as the kidney, small intestine, heart, and testicles, and to a lesser extent in the lung, liver, colon, spleen, muscle, blood, and brain (Li et al., 2020). Moreover, a low but constant expression of ACE2 has been revealed *via* the use of transcriptomic techniques on various brain structures, such as the brainstem, cortex, striatum, hypothalamus, choroid plexuses, and the paraventricular nuclei of the hypothalamus (Xia and Lazartigues, 2008; Chen R. et al., 2020). Given the evidence for the distribution of ACE2 in the brain, it can be inferred that multiple regions may be affected during SARS-CoV-2 infection. Furthermore, SARS-CoV-2 has a higher affinity for ACE2 than SARS-CoV and therefore, could have a major detrimental effect on the brain (Natoli et al., 2020).

**FIGURE 2:**
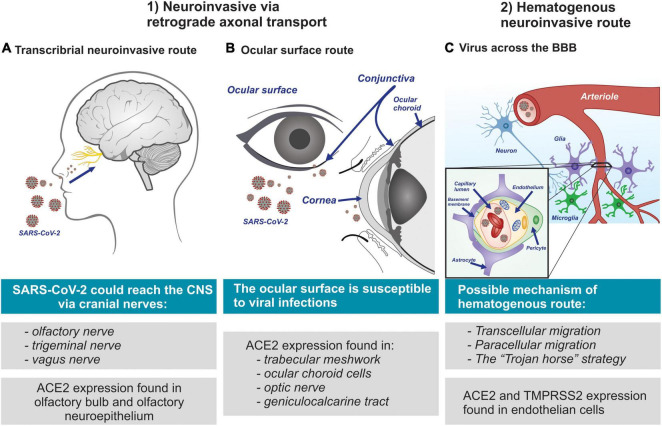
Mechanisms suggested through which SARS-CoV-2 invades the nervous system. **(A)** Transcribrial neuroinvasive route; SARS-CoV-2 may invade the brain through the olfactory nerve, and *via* cranial nerves. **(B)** Route ocular surface; multiple cell types of the visual system are suggested provide as potential entry points for SARS-CoV-2 invasion to the brain. **(C)** Hematogenous neuroinvasive route; SARS-CoV-2 possibly infects vascular endothelial cells *via* the ACE2 and TMPRSS2 receptors. Viral particles can reach the brain through the BBB by infecting and replicating inside brain microvascular endothelial cells. Moreover, SARS-CoV-2 infection can cause excessive peripheral immune responses to result in BBB dysfunction *via* cytokine storm.

The clinical severity of COVID-19 has been correlated with the frequency of neurological complications, while meningitis and encephalitis have been associated with paranasal sinusitis and could, in severe cases of SARS-CoV-2 infection, be an indicator of an aggravated viral infection due to an obstruction in the paranasal lymphatic vessels (Bridwell et al., 2020; Moriguchi et al., 2020). On the other hand, the glymphatic system, a glia-dependent elimination pathway for soluble wastes and metabolic products in the brain, is believed to play an important role in paranasal sinusitis. Serving as the brain’s “front end,” the glymphatic system is interconnected with the lymphatic network of the dura, cranial nerves, and veins of the skull (Benveniste et al., 2019). This interconnection could be used by SARS-CoV-2 to gain access to the brain in order to be internalized by the neurons. A compromised blood-brain barrier (BBB) and the perforation of the ethmoid bone are other suggested routes *via* which the virus enters the brain (Zubair et al., 2020; Pacheco-Herrero et al., 2021). Overall, three main routes for viral entry to the CNS have been proposed: (1) the transcribrial neuroinvasive route; (2) the neuroinvasive route *via* the ocular surface; and (3) the hematogenous neuroinvasive route (Figure 2). These proposed routes are discussed in the following sections.

### Evidence of Neuroinvasive Routes *via* Retrograde Axonal Transport

It has been proposed that one of the main neuroinvasive pathways into the CNS by SARS-CoV-2, as well as other viruses is *via* tropism, where a virus can infect a distinct group of cells by binding to the virus receptors on the surface of the host cell (Bauer et al., 2022a). In fact, chronic viral infections can be a risk factor for neurodegenerative diseases. In this regard, potential involvement of Japanese encephalitic virus, the influenza virus, herpes simplex virus type-1 (HSV-1) and the human immunodeficiency virus (HIV) in etiology of Parkinson’s disease and for Alzheimer’s disease have been suggested (De Chiara et al., 2012; Limphaibool et al., 2019; Sait et al., 2021). It is worth noting that HSV-1 infections are common, and that the virus can persist in a latent form within the neurons of its human host. Therefore, viruses may cause damage in vulnerable brain areas leading to neurodegenerative disease (Lotz et al., 2021; Sait et al., 2021).

#### Transcribrial Neuroinvasive Route of Severe Acute Respiratory Syndrome Coronavirus 2

It is suggested that SARS-CoV-2 reaches the olfactory bulb *via* infection of the peripheral nerve terminals of the olfactory sensory neurons (Mori, 2015; Desforges et al., 2019; Paniz-Mondolfi et al., 2020; Kumar et al., 2021). This proposed transcribrial route is supported by reports of SARS-CoV-2 patients presenting symptoms of anosmia and dysgeusia (Klopfenstein et al., 2020; Levinson et al., 2020; Mutiawati et al., 2021). It is noteworthy that ACE2 protein is highly expressed in the olfactory bulb (Brann et al., 2020) and the olfactory neuroepithelium (Sungnak et al., 2020; Ziegler et al., 2020). Thus, it is postulated that SARS-CoV-2 forms complexes with TMPRSS2 and neuropilin-1 *via* interaction with olfactory neuroepithelial ACE2, allowing it to enter into the brain retrogradely *via* the cranial nerves (Reza-Zaldívar et al., 2020; Messlinger et al., 2021). Indeed, there are reports that when some viruses infect the nociceptive neurons of the nasal cavity, they are able to reach the CNS through the trigeminal nerve (Deatly et al., 1990; Lochhead and Thorne, 2012) and other sensory terminals of the vagus nerve (Driessen et al., 2016). Hence, SARS-CoV-2 could follow the cranial nerves (Bulfamante et al., 2021; Messlinger et al., 2021) from their origin in the nasal cavity to the olfactory nerve, and then to the olfactory bulb, and finally arriving at the brain stem (Bougakov et al., 2021; Figure 2A). This pathway is also followed by the OC43 coronavirus strain (Dubé et al., 2018).

#### Neuroinvasive Route of Severe Acute Respiratory Syndrome Coronavirus 2 *via* the Ocular Surface

Recent studies conducted on both humans (Zhou et al., 2020; Collin et al., 2021) and mice (Ma et al., 2020) have demonstrated the expression of ACE2 and TMPRSS2 receptors in ocular surface cells, a region comprising the epithelial cells of the cornea and conjunctiva. Furthermore, a remarkable level of ubiquity of ACE2 receptor expression was recently found in the trabecular meshwork and ocular choroid cells of the outer eye, the optic nerve, and optic radiation or geniculocalcarine tract to the occipital cortex, which suggests that multiple cell types of the visual system provide potential entry points for SARS-CoV-2 invasion (Hill et al., 2021). However, as ACE2 expression has been observed more in the conjunctiva than in the cornea, SARS-CoV-2 has greater neuroinvasive potential *via* the conjunctiva (Leonardi et al., 2020; Ma et al., 2020). For the ocular surface to be considered a SARS-CoV-2 infection route, TMPRSS2 must be co-expressed along with ACE2, as TMPRSS2 acts as a cofactor in internalization of the complex (de Freitas Santoro et al., 2021). It should be noted that TMPRSS2 expression has been observed in both the cornea and the conjunctiva (Leonardi et al., 2020; Collin et al., 2021). However, the likelihood of the ocular surface being an infection gateway is low despite the potential of SARS-CoV-2 causing conjunctivitis and other ocular discomfort (Chen X. et al., 2021). However, the actual conjunctival transmission of SARS-CoV-2 is yet to be confirmed (de Freitas Santoro et al., 2021).

The ocular surface is susceptible to viral infections by means of aerosols or direct contact with fomites resulting from exposure to external contaminants and is vulnerable to a higher level of exposure than the oral cavity or the nostrils (Coroneo, 2021; Figure 2B). In addition, clinical cases where conjunctivitis was the initial symptomatic manifestation in COVID-19 positive patients have been reported and confirmed *via* both nasopharyngeal swab samples and PCR test which detected the presence of SARS-CoV-2 RNA in tears (Zhang X. et al., 2020; Hassan et al., 2021). Nonetheless, confirmation of the conjunctival transmission of SARS-CoV-2 into the CNS requires further investigation.

### Evidence of a Hematogenous Neuroinvasive Route of Entry for Severe Acute Respiratory Syndrome Coronavirus 2 to the Brain

The BBB tightly regulates the movement of molecules, ions, and cells between blood and the CNS and prevents the neurotoxic components of plasma, blood cells, and pathogens from entering the brain (Montagne et al., 2017). This regulatory characteristic is attributed to the arteries, arterioles, and capillaries that supply blood to the brain and that act in response to neuronal stimuli that trigger an increased rate of neurovascular coupling, a mechanism generated by the cerebral blood flow and the supply of oxygen. The neurovascular unit is made up of the following structural components: vascular cells (endothelium and wall cells, pericytes, and smooth muscle cells); glia (astrocytes and microglia); and neurons (Kisler et al., 2017; Sweeney et al., 2019; Sanchez-Cano et al., 2021). The regulation conducted by the BBB provides strict control over the cellular permeability of neuronal tissues, which is essential for proper neuronal function and which, furthermore, requires precise ionic concentrations in the surrounding environment (Daneman, 2012; Daneman and Prat, 2015). Therefore, the loss of homeostatic regulation and deterioration in the restrictive capacity of the BBB play important roles in the progression of neurological conditions such as brain trauma, and infectious and neurodegenerative diseases (Sanchez-Cano et al., 2021).

It is of particular interest to note that the ACE2 and TMPRSS2 receptors are expressed in the endothelial cells of the BBB (Chen R. et al., 2020; Qiao et al., 2020; Torices et al., 2021). Due to these findings and the interaction of the virus with the protein complex discussed earlier in this paper, it has been suggested that SARS-CoV-2 could reach the brain *via* systemic circulation by crossing the BBB and damage the choroid plexus (Baig, 2020; Pellegrini et al., 2020). The actual hematogenous mechanism by which SARS-CoV-2 gains entry to brain is not known. However, several mechanisms have been suggested (Achar and Ghosh, 2020; Pellegrini et al., 2020). These include: (a) transcellular migration, where the virus invades the host’s endothelial cells and is able to cross the BBB; (b) paracellular migration, where the virus invades the choroid plexus of the fenestrated endothelial cells and gets into the brain; and (c) the “Trojan horse” strategy, where the virus is internalized by phagocytic immune cells such as neutrophils and macrophages, and is subsequently replicated in the brain (Dahm et al., 2016; Figure 2C). Moreover, is likely that SARS-CoV-2 invades the brain by damaging the integral architecture of the BBB (Varghese et al., 2020). Thus, SARS-CoV-2 can get access into the brain by one or a combination of the above mechanisms.

### Evidence of Neuroinvasive Mechanisms of Severe Acute Respiratory Syndrome Coronavirus 2 in Animal Models

Both the symptoms presented by infected patients and the findings of clinical pathology provide evidence of possible infection of the CNS by SARS-CoV-2. However, to further explore the viral pathogenesis in the host and characterize the mechanisms of viral access and dissemination in the CNS, a translational neuroscience approach (Johansen et al., 2020; Sanclemente-Alaman et al., 2020), similar to that employed in the early research conducted on SARS and MERS is necessary (Callaway, 2020; Natoli et al., 2020).

Because SARS-CoV-2 has a higher affinity for the human ACE2 receptor (hACE2) than animal ACE2, few studies have been carried out in animal models to determine the neuroinvasive pathways of the virus (Wan et al., 2020). In addition, hACE2 is structurally different from that in animal species. Hence, a significantly lower level of tropism is noted in animal vs. human tissue, particularly in relation to CNS (Natoli et al., 2020). Indeed, Brann et al. (2020) demonstrated that the olfactory sensory neurons of the whole olfactory mucosa of mice, unlike olfactory epithelial support cells, stem cells, and the cells of the nasal respiratory epithelium, do not express ACE2 and TMPRSS2 genes. Thus, it is argued that based off of animal studies, anosmia or other forms of olfactory dysfunction may not support olfactory bulb as an entry route for SARS-CoVs into the CNS (Brann et al., 2020; Natoli et al., 2020).

To overcome the discrepancy between animal and human studies, several animal models with closer resemblance to that of humans have been suggested. One suggestion is to develop humanized mouse models that express the hACE2 receptor (Sun et al., 2020), as the murine is the most widely used animal model for this purpose (Muñoz-Fontela et al., 2020). Another suggestion is to modify the SARS-CoV-2 spike to effectively bind with murine-ACE2 (Dinnon et al., 2020), however, this strategy is risky, since modifying the viruses can create a natural reservoir for a virus that might be completely different from the wild-type version. A third suggestion is to induce mice to be susceptible to SARS-CoV-2 infection by sensitizing the respiratory tract to the virus. The latter may be achieved *via* transduction with adenovirus or associated viruses that express hACE2 (Ad5-hACE2 or AAV-hACE2, respectively) (Israelow et al., 2020; Rathnasinghe et al., 2020). In all these suggestions, however, as mentioned earlier, it must be borne in mind that the co-expression of the ACE2 and TMPRSS2 receptors is necessary for the virus to be internalized.

It was recently demonstrated that neuroinvasion by SARS-CoV-2 could be achieved in an animal model where hACE2 was overexpressed by means of an adeno-associated virus infection (Song et al., 2021). Moreover, the neuronal infection could be prevented by blocking ACE2 with neutralizing antibodies or administering cerebrospinal fluid obtained from a COVID-19 patient, where presumably antibodies were present (Song et al., 2021). A recent study reported differences in the neuroinvasiveness and neurovirulence among the most relevant SARS-CoV-2 variants, D614G, Delta (B.1.617.2), and Omicron (B.1.1.529) 5 days post inoculation in a hamster model. The results showed that D614G variant had a high neuroinvasion *via* the olfactory nerve compared to the Delta and Omicron variants (Bauer et al., 2022b). While the results obtained provide evidence for the neuroinvasive capacity of SARS-CoV-2 in an animal model, the sequence of infection in different CNS cell types has not yet been determined. Therefore, more studies on detailed mechanism (s) of SARS-CoV-2 infection of the CNS are needed.

## Neuropathological Features of Severe Acute Respiratory Syndrome Coronavirus 2

SARS-CoV-2 has been reported to manifest neurological symptoms that range from mild to fatal, while it can also occur asymptomatically in patients. Clinical studies conducted on patients hospitalized with COVID-19 report a level of neurological manifestation ranging from 15.2% (Flores-Silva et al., 2021), or 36.4% (Mao et al., 2020) to 54.7% (Romero-Sánchez et al., 2020), and up to 88% (García-Moncó et al., 2021). It should be noted that the frequency of neurological alterations observed in patients with COVID-19 depends on whether they have been evaluated by a neurologist and/or inclusion of patients with a history of neurological complication.

The most common early neurological manifestations in patients with COVID-19 are headache, dizziness, nausea, vomiting, myalgia, and neuralgia (Guan et al., 2020; Mao et al., 2020; Wang et al., 2020; Song et al., 2021). Anosmia and dysgeusia develop in the early stages of infection and are more frequent in less severe cases (Mao et al., 2020; Flores-Silva et al., 2021; García-Moncó et al., 2021). Late-infection neurological manifestations include acute cerebrovascular disease, meningoencephalitis, impaired consciousness, and skeletal muscle injury (Al Saiegh et al., 2020; Guan et al., 2020; Guidon and Amato, 2020; Parikh et al., 2020). Less-frequently reported symptoms include dysautonomia, seizures, movement disorders, Guillain Barré syndrome, Miller Fisher syndrome, and optic neuritis (Gutiérrez-Ortiz et al., 2020; Hwang et al., 2020; Manji et al., 2020). In addition, the University College London Queen Square Institute of Neurology has reported five categories of clinical presentations at a neurological level: (1) encephalopathy with delirium/psychosis and no magnetic resonance imaging or cerebrospinal fluid abnormalities; (2) inflammatory CNS syndromes including encephalitis and acute disseminated encephalomyelitis; (3) ischemic strokes; (4) peripheral neurological disorders including Guillain–Barré syndrome and brachial plexopathy; and (5) miscellaneous central nervous disorders (Paterson et al., 2020; Gasmi et al., 2021). Thus, neurological manifestations are variable and not uncommon in COVID-19 patients. Moreover, both morphological and biochemical pathological changes may be manifested as detailed below.

### Morphological Changes

Research on the clinical and imaging aspects of COVID-19 infection as well as molecular biology studies conducted on both *in vitro* and *in vivo* models have provided valuable information in understanding the etiological mechanisms of SARS-CoV-2. However, despite the large amount of information available on the disease, there is little work conducted to characterize its pathological manifestations in the tissues of different systems of the body (Al Nemer, 2020; Skok et al., 2021; Table 1). Studies carried out on the anatomical brain pathology during autopsy reveal morphological alterations in the frontal and occipital lobes, olfactory bulb, cingulate gyrus, corpus callosum, hippocampus, basal ganglia, thalamus, cerebellum, midbrain, middle pons, medulla, brainstem, and the lateral ventricles (Barone et al., 2021; Caramaschi et al., 2021; Generoso et al., 2021). The most common gross findings are edema (Reichard et al., 2020), hemorrhagic lesions (Paniz-Mondolfi et al., 2020; Reichard et al., 2020), hydrocephalus (Lacy et al., 2020), atrophy and low brain mass (Lax et al., 2020; Wichmann et al., 2020), olfactory bulb asymmetry (Coolen et al., 2020), and infarcts (Solomon et al., 2020). SARS-CoV-2 has also been found to cause lesions and alterations in neuronal structures, while neuronal infection can cause encephalitis and the generation of lethal microthrombi (Bradley et al., 2020; von Weyhern et al., 2020). In addition, severe COVID-19 infection accompanied by multisystem inflammatory syndrome may cause fibrotic lesions and generate cerebral thrombosis (Turski et al., 2020).

**TABLE 1:** CNS damage by SARS-CoV-2 suggesting susceptibility to Parkinson’s and Alzheimer’s disease.

Characteristics	Alzheimer’s disease	Parkinson’s disease	References
Affected brain regions (Autopsy findings)	Hippocampus, cortex (Frontal, occipital, and cingulate), insula.	Midbrain, basal ganglia, thalamus.	Solomon et al., 2020; von Weyhern et al., 2020; Generoso et al., 2021
Brain morphological and macroscopic changes	Edema, hemorrhagic lesions, hydrocephalus, atrophy and low brain mass, infarcts.		Paniz-Mondolfi et al., 2020; Reichard et al., 2020; Wichmann et al., 2020
Brain regions expressing ACE2 and TMPRSS2	Prefrontal cortex, hippocampus.	Striatum (Human brain), SNpr, SNpc (mouse brain)	Chen R. et al., 2020; Qiao et al., 2020
Intracellular SARS-CoV-2	Frontal cortex	No findings report	Paniz-Mondolfi et al., 2020; Liu J. M. et al., 2021
Biochemical markers	Glia and microglia response, cytokine storm (increased interleukins IL-1β, IL-6, IL-8, IL-12, IL-17, IL-18). Oxidative stress in various brain regions such as BBB and neurons susceptible to cell death.		Chaudhry et al., 2020; Almutairi et al., 2021;Chiricosta et al., 2021; Frank et al., 2022

*SNpr, substantia nigra pars reticulate; SNpc, substantia nigra pars compacta; IL, interleukin.*

Various studies have been carried out to ascertain the structural modifications in the brains of COVID-19 patients. Magnetic resonance techniques have shown a bilateral obliteration of the olfactory cleft in 50% of SARS-CoV-2-positive patients as well as a sudden loss of smell and subtle olfactory bulb asymmetry in 25% of the sampled patients (Niesen et al., 2021). Another study demonstrated microstructural changes using diffusion tensor imaging (DTI) and 3D high-resolution T1WI sequences in COVID-19 patients, where greater volume of bilateral gray matter (reported as gray matter volume) was observed in the hippocampus, olfactory cortices, insula, left rolandic operculum, left Heschl gyrus, and right cingulate gyrus (Lu Y. et al., 2020). These findings demonstrate possible alterations in the structural and functional integrity of brain microstructures in susceptible patients, and also suggest potential long-term consequences of SARS-CoV-2 infection, which may lead to or accelerate a variety of neurodegenerative diseases (ElBini Dhouib, 2021), discussed further below.

### Molecular and Biochemical Changes

Given that most cases of SARS-CoV-2 infection present mild or moderate symptoms and that a group with severe infections develops multiple systemic dysfunctions as a consequence of imbalances in the immune and the oxidation-reduction systems, it is not surprising that inflammatory states and oxidative stress are commonly indicated in pathogenesis of COVID-19 (Mrityunjaya et al., 2020; Forcados et al., 2021). In addition, hyperactivation of the immune system leading to an exaggerated release of pro-inflammatory cytokines referred to as “cytokine storm” is not only associated with severe complications, but also poorer outcome (Chen G. et al., 2020; Noroozi et al., 2020; Tan et al., 2021; Yang et al., 2021).

Both the innate and adaptive immune systems have been widely described as working together, with the innate response representing the host’s first line of defense and the adaptive response becoming prominent several days after infection, when T and B cells have undergone clonal expansion (Strbo et al., 2014). Furthermore, the components of the innate system contribute to the activation of antigen-specific cells, which amplify their responses in order to achieve complete control over the pathogen by recruiting innate effector mechanisms. Therefore, the innate and adaptive responses are fundamentally different, although the synergy between them is essential for an effective immune response (Chaplin, 2010). Patients with severe SARS-CoV-2 infection manifest an increased innate immune response and a suppressed adaptive immunity, which is why a delayed elimination of the virus from the organism is observed (Pan Y. et al., 2021). This scenario aggravates the immune status of the patient as it increases the levels of various inflammatory factors and increases both the number and activation of immune cells at the site of the inflammation, a process from which cytokine storm originates (Catanzaro et al., 2020; Chen R. et al., 2021).

Neurons infected by SARS-CoV-2 release inflammatory mediators that are capable of activating adjacent cells such as glia, microglia, mast cells and endothelial cells, conditions which constitute the beginning of neuroinflammation (Almutairi et al., 2021; Frank et al., 2022). Elevated immunological mediators during SARS-CoV-2 infection include: tumor necrosis factor α (TNF-α); interferon gamma (INFγ); a series of chemokines such as CCL-2, CCL-5, and CXCL-10; a series of interleukins such as IL-1β, IL-6, IL-8, IL-12, IL-17, IL-18, and IL-33; and granulocyte macrophage colony stimulating factor (GM-CSF) (Kempuraj et al., 2020; Mehta and Fajgenbaum, 2021; Tripathy et al., 2021). SARS-CoV-2 infection induces the down-regulation of ACE2, disrupting the physiological balance between ACE/ACE2 and angiotensin II (Ang-II)/angiotensin and leading to severe multiple organ injury (Mehrabadi et al., 2021). In fact, it has been suggested that ACE2 downregulation may contribute to the pathogenesis of lung injury in COVID-19 (Ni et al., 2020). Angiotensin II stimulates gene expression of multiple inflammatory cytokines such as TNF-α and IL-6. TNF-α, in particular, induces macrophage differentiation of a pro-inflammatory phenotype, which exerts an antimicrobial effect. However, such differentiated macrophages are also responsible for recruiting more cell types *via* cytokine secretion, thus exacerbating the inflammatory response. Similarity, IL-6, essential for T cell differentiation, when elevated, signals a poorer SARS-CoV-2 prognosis (Banu et al., 2020; Patra et al., 2020; Ben Moftah and Eswayah, 2022).

Additionally, the pro-inflammatory factors discussed above are able to cross the BBB, increase vascular permeability, and trigger further release of pro-inflammatory cytokines from the microglia (da Fonseca et al., 2014; Zhang et al., 2021). This cascade results in increased apoptotic activity, increased levels of reactive oxygen species (ROS), mitochondrial dysfunction and eventual neurodegeneration (Chaudhry et al., 2020; Chiricosta et al., 2021; Kumar et al., 2021). Moreover, the high concentration of pro-inflammatory cytokines can lead to the activation of the coagulation cascade, suppression of anticoagulant factors, and hence increase the chance of thrombosis (Al Saiegh et al., 2020; Levi et al., 2020). An opposite scenario may also manifest itself where an increase in fibrinolytic activity leading to an increase in the level of fibrin degradation and hemorrhagic conditions including aneurysms may be observed in certain patients infected with SARS-CoV-2 (Al Saiegh et al., 2020).

## Severe Acute Respiratory Syndrome Coronavirus 2 and Neurodegenerative Disease

**FIGURE 3:**
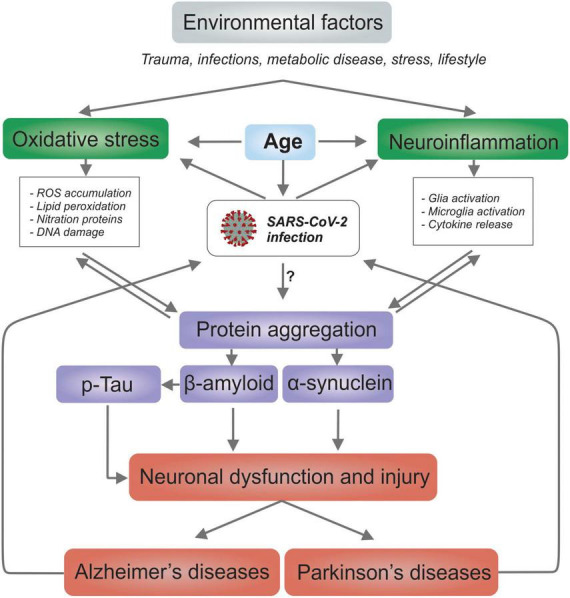
SARS-CoV-2 infection and possible links for both Alzheimer’s and Parkinson’s disease. The common risk factors between COVID-19 and neurodegenerative diseases is the age; the oxidative stress; and, neuroinflammation. It’s possible that COVID-19 patients are more susceptible to developing neurodegenerative diseases and that patients with neurodegenerative diseases are more susceptible to contracting COVID-19.

The link between systemic and central inflammation, as well as between neurological and neuropsychiatric diseases, is well known (Hurley and Tizabi, 2013; Schwartz and Deczkowska, 2016; Skaper et al., 2018). In nervous tissue, the increased levels of inflammatory mediators and glial cell activity caused by SARS-CoV-2 infection may pose an increased risk of neurodegenerative disease such as Alzheimer’s disease (AD), Parkinson’s disease (PD) as well as multiple sclerosis (MS), stroke and neurological trauma (Mohammadi et al., 2020; Li et al., 2021; Tekiela and Majersik, 2021). SARS-CoV-2 infection in people with senility is likely to increase the neuropathological intensity and contribute to the worsening of motor and cognitive deficits (Wang Y. et al., 2021; Yu et al., 2021). Indeed, one of the main risk factors for both COVID-19 and neurodegenerative disease is age (Ferini-Strambi and Salsone, 2021; Figure 3). Older people comprise the section of the population most prone to developing neurodegenerative diseases (Hou et al., 2019) and present with more severe clinical reaction to COVID-19 (Lebrasseur et al., 2021). In addition, lifestyle and preexisting conditions such as trauma, infection, metabolic disease, and stress can accelerate the onset and progression of neurodegenerative diseases (Graham and Sharp, 2019; Madore et al., 2020; Lotz et al., 2021).

It is important to note that protein aggregation in the brain is considered as one of the main reasons behind neurodegeneration. Protein aggregation has been observed for tau protein and Aβ peptide in AD; and α-synuclein for PD. The aggregation process spreads from one cell to another, and the aggregates and deposits formed impair brain function. It is unclear whether viral infections directly or indirectly cause neurodegeneration (Liu S. et al., 2021). However, it has been suggested that viruses can initiate pathological protein aggregation *via* a direct mechanism in Aβ peptide aggregation *in vitro* and in animal models infected with HSV-1 and respiratory syncytial virus (Ezzat et al., 2019). A recent study has shown direct interactions between the N-protein of SARS-CoV-2 and α-synuclein as the molecular basis for the observed correlation between SARS-CoV-2 infections and parkinsonism (Semerdzhiev et al., 2022). Given that cell-surface glycans function as initial, usually low-affinity attachment factors, these receptors play key roles in SARS-CoV-2 infection (Koehler et al., 2020; Prasad et al., 2021). Moreover, the ability of S1 protein of SARS-CoV-2 to interact with heparin receptor can lead to many misfolded brain proteins including amyloid complex and ultimately lead to neurodegeneration (Tavassoly et al., 2020; Idrees and Kumar, 2021).

### Alzheimer’s Disease

One of the main causes of disability among older people around the world is dementia, with AD as the most common form (Alzheimer’s-Association, 2020). The mechanisms involved in the pathogenesis of AD are complex and not fully understood. However, the most accepted hypothesis involves molecular changes such as extracellular deposition of the β-amyloid protein and intracellular phosphorylation of the tau protein, causing the formation of amyloid plaques and neurofibrillary tangles, respectively (Liu et al., 2019). In addition, intense neuroinflammation, oxidative stress, mitochondrial dysfunction, and protein misfolding have also been implicated (Gandhi et al., 2019; Perez Ortiz and Swerdlow, 2019; Leng and Edison, 2021; Figure 3). Patients with AD are more susceptible to contracting COVID-19 (Finelli, 2021; Pan A. P. et al., 2021; Wang Q. et al., 2021), and COVID-19 patients are more susceptible to developing AD (Brown et al., 2020; Chiricosta et al., 2021; Villa et al., 2022). This might not be surprising due to the presence of common risk factors such as age, cardiovascular disease, metabolic, and psychological disorders between the two diseases (Brown et al., 2020; Finelli, 2021). Generally, inflammation increases with age, wherein higher levels of pro-inflammatory cytokines have been quantified in older people (Niraula et al., 2017; Rea et al., 2018). Furthermore, given that infection with a wide variety of pathogens is suspected to be a risk factor for the onset of AD (Seaks and Wilcock, 2020; Vigasova et al., 2021), an increased risk of developing AD and cognitive impairment in susceptible populations after SARS-CoV-2 infection would also be expected. Moreover, these patients often face social stigma and mental stress, which can further aggravate neuroinflammatory processes and result in psychiatric disorders (Justice, 2018; Milligan Armstrong et al., 2021). The presence of pathogens and other factors such as age, alcohol and tobacco consumption, cerebral hypoxia, metabolic diseases, pollution, sedentary lifestyle, or sleep disorders may cause BBB malfunction (Noe et al., 2020; Hussain et al., 2021), and hence lead to the infiltration of neurotoxic proteins such as the β-amyloid peptide (Wang D. et al., 2021). SARS-CoV-2 infection accompanied by a local immune response incorporating astrocytes and microglia could generate a state of neuroinflammation in susceptible patients that can manifest for a long term (Kumar et al., 2021; Villa et al., 2022). Such a scenario would be expected to exacerbate the current pathology in AD patients.

Genetically, apolipoprotein E ε4 allele (APOE4) has been determined the strongest risk factor for AD (Serrano-Pozo et al., 2021). Furthermore, APOE4 has been associate with increased susceptibility to SARS-CoV-2 infection and COVID-19 (Kurki et al., 2021). Cerebral microvasculature complications may be the basis of neurological issues in hospitalized COVID-19 patients (Miners et al., 2020; Lee et al., 2021). Interestingly APOE4 is also involved in BBB dysfunction and cerebrovascular diseases (Montagne et al., 2021). However, further research is needed to determine the exact role of APOE4 in COVID-19-induced AD.

### Parkinson’s Disease

Similar to AD, PD is related to COVID-19, sharing risk factors such as advanced age, cardiovascular and metabolic diseases (Park et al., 2020; Fearon and Fasano, 2021; Sharifi et al., 2021). Moreover, patients with PD may be immunosuppressed, which makes them more susceptible to infections of any type including SARS-CoV-2 (Tansey and Romero-Ramos, 2019; Prasad et al., 2020).

Histologically, PD is characterized by the loss of dopaminergic neurons in the *substantia nigra pars compacta* (SNpc) and cytoplasmic inclusions, mainly composed of α-synuclein aggregates called Lewy bodies (Henderson et al., 2019; Figure 3). Although the precise etiology of PD is not well known, some hypotheses for its pathogenesis and development point to oxidative stress, neuroinflammation, mitochondrial dysfunction, synaptic pathogenesis, and also as a result of infection (Hurley and Tizabi, 2013; Meng et al., 2019). As patients in an advanced stage of PD have difficulties in chewing and swallowing, they commonly experience salivary accumulation and aspiration (Kwon and Lee, 2019). In addition, the stiffness of the chest wall common in PD inhibits the cough reflex, forming a favorable environment for SARS-CoV-2 infection. In the most severe cases, SARS-CoV-2 infection progresses to pneumonia, which is one of the leading causes of death in PD patients (Bhidayasiri et al., 2020). It is already well known that various viral infections can accentuate the pathological sequelae of PD (Jang et al., 2009).

Recently, a study demonstrated the ability of the H1N1 influenza virus to block protein degradation pathways and promoting the formation of α-synuclein aggregates in dopaminergic neurons *in vitro* (Marreiros et al., 2020), and a more recent study provided similar results in an *in vivo* model (Bantle et al., 2021). Furthermore, increased amounts of phosphorylated α-synuclein, activation of microglia and astrocytes, and selective loss of dopaminergic neurons in the SNpc with behavioral and motor consequences have been observed as secondary consequences of infection with the western equine encephalitis virus (WEEV) (Bantle et al., 2019). Thus, it is not unreasonable to expect similar consequences with SARS-CoV-2 infection (Chaná-Cuevas et al., 2020). Moreover, COVID-19 pandemic causing at least partial confinement and social distancing may not only limit the mobility but may also aggravate depression, mental stress, and loneliness in PD patients. It is noteworthy that there exists a substantial co-morbid condition of PD and depression (Tizabi et al., 2019, 2021).

## Immediate Prospects

In this article, we have reviewed the possible routes by means of which SARS-Cov-2 may enter the CNS, including transcribrial or the ocular surface, and the hematogenous neuroinvasive pathways. The increased occurrence of neuroinflammation, oxidative stress, and cytokine storm caused by the virus also increases the level of cellular damage in the CNS, thus increasing the risk of developing neurodegenerative diseases such as AD and PD. Further studies on effects of SARS-CoV-2 and its variants on protein aggregation, glia or microglia activation, BBB damage, oxidative stress, and neuroinflammation is warranted.

## Author Contributions

JS, FP, AP-M, and IL designed the sections and contents of the review article. IL and FP oversaw the organization to distribute the writing tasks among the authors and participated in article writing. FP made all the figures. JS, FP, AP-M, GS-L, LC, YT, and IL performed literature search and participated in the article writing. All authors critically reviewed and approved the final version of the article.

## Conflict of Interest

The authors declare that the research was conducted in the absence of any commercial or financial relationships that could be construed as a potential conflict of interest.

## Publisher’s Note

All claims expressed in this article are solely those of the authors and do not necessarily represent those of their affiliated organizations, or those of the publisher, the editors and the reviewers. Any product that may be evaluated in this article, or claim that may be made by its manufacturer, is not guaranteed or endorsed by the publisher.
